# Ganoderma terpenoid extract exhibited anti-plasmodial activity by a mechanism involving reduction in erythrocyte and hepatic lipids in *Plasmodium berghei* infected mice

**DOI:** 10.1186/s12944-018-0951-x

**Published:** 2019-01-12

**Authors:** Olarewaju M. Oluba

**Affiliations:** Department of Biological Sciences, Food Safety and Toxicology Research Unit, Environment and Technology Research Cluster, College of Science and Engineering, Landmark University, P.M.B, Omu-Aran, Kwara State 1001 Nigeria

**Keywords:** Ganoderma lucidum, Terpenoid extract, Antiplasmodial activity, Hypolipidemic activity, Hepatoprotection

## Abstract

Bioactive components of *Ganoderma lucidum* has recently gained intense research attention due to their acclaimed nutritional and medicinal properties. Thus, the terpenoid extract from the fruit bodies of *G. lucidum* (GT) was evaluated for activity against *Plasmodium berghei* in mice in two separate experiments. In addition, the effects of the extract on erythrocyte and hepatic lipids as well as liver HMG-CoA reductase activity before and after the treatments were also assessed. Mice with established infection were administered 100 and 250 mg/kg/day GT alone and in combination with chloroquine (CQ), in either case two separate controls designated: CQ (30 mg/kg chloroquine) and INF-CTR (1 mL DMSO) were also included. Treatment was administered orally for 12 days and parasitemia determined every three days. Percentage survival was significantly increased to 87% from 66% due to combination of GT100 with CQ compared to GT100 alone and to 75% from 62% when GT250 was administered with CQ compared to GT250 alone. Erythrocyte triglycerides, total cholesterol (TC), LDL and phospholipids contents were significantly lower in GT + CQ-treated mice compared to CQ alone and INF-CTR. Similarly, hepatic TC and phospholipid levels were significantly lower in the GT + CQ-treated mice compared to CQ alone and INF-CTR and HMG-CoA reductase activity in the liver was significantly inhibited due to administration of GT + CQ. Data from this study suggest that the anti-plasmodial action of GT could involve mechanisms associated with its hypolipidemic activity. It was also demonstrated that chloroquine, when administered in combination with GT, potentiates its curative effect in *P. berghei-infected* mice.

## Introduction

Malaria remains a major public health challenge affecting 3.3 billion people in 106 countries and territories. About 216 million malaria cases are reported to occur annually 81% of which occur in the African sub-region [1]. Out of about 438,000 deaths resulting from malaria in 2015, an estimated 90% were traced to sub-Saharan Africa [1]. Nigeria with about 51 million cases and 207,000 deaths contributes approximately 30% of African annual malaria burden. According to a WHO report about 97% of the total Nigerian population (approximately 175 million) is at risk of malaria infection [1].

The rising problem of resistance to available chemotherapies and problem of recrudescence of malaria after treatment with artemisinin stress the need for new antimalarial agents [2]. The classical drugs quinine and artemisinin are both plant derivatives obtained from Cinchona specie [3] and *Artemisia annua* [4] respectively, suggesting that other effective drugs might be plant-derived. Plants and fungi products have formed a major component of foods and therapeutic agents of indigenous African communities for centuries. In recent times, increased research attention has focused on phytochemicals extracted from plants with traditionally acclaimed medicinal properties in order to elucidate the scientific basis for their observed activities and to seek new lead compound from them which could be developed into therapeutic drugs with enhanced efficacy and minimal side effects [5].

The rationale for the search of antimicrobial compounds from fungi is that both fungi and humans share common microbial pathogens such as *Escherichia coli* and *Staphylococcus aureus*, therefore we can benefit from their defense strategies against these microorganisms [6]. There has been paucity of information on African Ganoderma and majority of medicinal investigations on Ganoderma species have been carried out on species that have been isolated from other parts of the world. Since species within the Ganodermataceae family all have a common evolutionary history, it is not unlikely that African Ganoderma may contain similar compounds to those isolated elsewhere.

*Ganoderma lucidum* is an edible mushroom with long history of use as a medicinal herb in Oriental countries [7]. The extract has been investigated for its health benefits in preventing cardiovascular disease, cancer, and microbial infection, and for its lipid and glucose lowering, anti-inflammation, anti-oxidant, anti-parasite, and multiple organ protection effects [8–15]. Its long history of usage with no attendant toxicity represents the desired end result in the development of effective therapeutic interventions.

In West Africa, many health claims have been made on the effect of *Ganoderma* species on immune system. Traditional medical practitioners usually consider *Ganoderma* as natural immune regulator [9]. Bioactive components of the medicinal mushroom, *Ganoderma lucidum* has recently gained intense research attention due to their acclaimed nutritional and medicinal properties. Experimental evidences revealed that the terpenoids are the most important biologically active substances in *Ganoderma lucidum* due to their varying pharmacological applications [8, 9]. Terpenoids extracted from Ganoderma lucidum have been demonstrated to exhibit anti-hypertensive [8], hypocholesterolemic [10, 11], hepatoprotective [12], and cytotoxicity against numerous cancer cell lines [13]. A recent study by Oluba et al. [15] demonstrated the promising potential of crude chloroform extract of *Ganoderma lucidum* fruit body as an anti-plasmodial agent.

Changes in erythrocyte lipid composition and the host oxidant-antioxidant status are two critical associated events involved in the pathogenesis of malaria [16]. During the intraerythrocytic stage of the parasite development, about 5 to 10 merozoites are produced every 24 h. A corresponding increase in metabolic activity with concomitant membrane turn-over rate is required to sustain the growing parasite at this stage. Labaied et al. [17] have shown that *Plasmodium falciparum* infection induces a six-fold increase erythrocyte phospholipid content. In order to meet up with its high requirement for phospholipids, the infected erythrocytes contain phospholipid synthesizing enzymes [18]. Thus, potent inhibitors of plasmodial phospholipid synthesis was previously characterized as potential target for anti-malarial chemotherapy due to its crucial role to the parasite survival [19].

The mechanism(s) contributing to the anti-plasmodial activity of *Ganoderma lucidum* extract remains unclear. Increasing evidence indicates that changes in tissue lipid content may be implicated in the process [12]. The present study was aimed at extracting the terpenoid fraction of *Ganoderma lucidum* fruit body and evaluation of its potential anti-plasmodial activity when administered alone or in combination with chloroquine against *Plasmodium beghei* in mice.

## Materials and methods

### Fungal material

*Ganoderma lucidum* fruit bodies were collected from a forest reserve at Ipele, Ondo State, Nigeria and botanically identified by Dr. Soji Fakoya, a mycologist in the Department of Biological Sciences, Joseph Ayo Babalola University, Ikeji-Arakeji, Nigeria. Specimen sample assigned voucher number 1103 was deposited in institution herbarium for reference purpose.

### Preparation of ganoderma terpenoid extract (GT)

*G. lucidum* fruit body thoroughly washed with clean sterile water was air-dried at room temperature for two weeks. The dried samples were cut into smaller pieces and milled into powder using a mechanical grinder. The terpenoid extraction was carried out according to the method described by Weng et al. [20] with little modifications. Briefly, the powdered fungal material (2000 g) was first extracted by reflux using 50% ethanol for 24 h at room temperature. The resulting mixture was filtered using Whatman number 1 filter paper and the combined filtrate concentrated in vacuo at 35 °C. The concentrated extract was partitioned between chloroform and water. The combined chloroform layer was extracted with 5% NaHCO_3_ solution. The combined NaHCO_3_ fraction was acidified to pH 3 with 2 N HCl under ice-cooling and then extracted with chloroform. The combined chloroform layer was evaporated under reduced pressure to give a mixture of acidic compounds herein referred to as Ganoderma terpenoid extract (GT). This was stored in dark airtight container at 4 °C until used for the experiment.

#### Quantification of total terpenoid content

Total terpene content of the GT extract was quantified spectrophotometrically based on the method described by Gao et al. [21] using ganoderic acid A as standard. The total triterpenoid content was expressed as milligrams of ganoderic acid A equivalents per gram of GT.

#### Animals and treatment

Eighty-four (84) *Swiss* male mice (between 7 and 9 weeks old) procured from the animal care facilities of Institute for Advanced Medical Research and Training (IAMRAT), University Teaching Hospital, Ibadan, Nigeria were used for the study. The animals were kept in standard mice cages under a controlled environment and with unrestricted access to food and water. Approval for the study was granted by Joseph Ayo Babalola University Research and Ethics Committee with approval number JABU/REC/AS015. All animal experimental procedures were carried out in strict compliance with the rules guiding the Care and Use of Laboratory Animals as contained in the National Institutes of Health manual [22].

#### Parasite inoculation

The malarial parasite *Plasmodium berghei* (NK-65 strain) was obtained from the Institute for Advanced Medical Research and Training (IAMRAT), UCH, Ibadan, Nigeria.

#### Ganoderma terpenoid extract treatment

Two sets of animal experiments were carried out. In the first experiment intended to ascertain the effect of GT terpenoid extract alone, 32 male mice were inoculated intraperitoneally (i.p.) with 0.2 mL 10^5^ *P. berghei*-infected red blood cells suspended in PBS. Three days after parasite inoculation, mice in each group were fasted overnight, weighed and their parasitemia determined. Thereafter, they were randomly assigned into four experimental groups (*n* = 8) designated: CQ, administered 30 mg/kg gavage chloroquine—hydroxychloroquine sulfate (Aspen Pharma, Johannesburg, South Africa); GT_100_, received 100 mg/kg Ganoderma terpenoid extract; GT_250_, received 250 mg/kg Ganoderma terpenoid extract; INF-CTR, received 1 mL DMSO. The extract was dissolved in DMSO in order to keep the concentration and dose similar. Treatment was administered once daily for 12 consecutive days during which they were observed daily for survival and their parasitemia level determined every three days. In another separate experiment, intended to ascertain the effect of GT extract administered in combination with CQ,52 male mice were inoculated intraperitoneally (i.p.) with 0.2 mL 10^5^ *P. berghei*-infected red blood cells suspended in PBS. Three days after parasite inoculation, mice in each group were fasted overnight, weighed and their parasitemia determined. Thereafter, they were randomly assigned into four experimental groups (*n* = 13) designated: CQ, administered 30 mg/kg gavage chloroquine—hydroxychloroquine sulfate; GT_100_ + CQ, received a combination of 100 mg/kg Ganoderma terpenoid extract and 30 mg/kg gavage chloroquine—hydroxychloroquine sulfate; GT_250_ + CQ, received a combination of 250 mg/kg Ganoderma terpenoid extract and 30 mg/kg gavage chloroquine—hydroxychloroquine sulfate; INF-CTR, received 1 mL DMSO. Before commencement of the extract treatment, five mice from each group were sacrificed and their blood samples collected and analyzed to obtain the baseline level of each parameter monitored. After 12-day treatment, the animals were fasted overnight, weighed and their parasitemia estimated from their tail blood samples before been euthanized and then sacrificed by cervical dislocation. Upon sacrifice, blood sample was collected from each animal into a plain sample bottles, liver, kidney, spleen and brain were quickly excise, blotted on tissue paper, cleared of fats and weighed.

### Serum preparation

Blood samples collected in plain tubes were left standing for 3 h to clot and then centrifuged at 5000 rpm for 10 min at 4 °C. Serum from the centrifuged blood sample was collected by suction using pasture pipette into sterile plain sample bottles and stored at 4 °C for further analysis.

### Tissue preparation

Liver homogenate was prepared by weighing 2 g of cleaned tissue and homogenized using 0.1 M phosphate buffer after which it was centrifuged at 5000 rpm (4 °C) for 10 min to obtain the supernatant which was subsequently used as tissue homogenate. Erythrocytes and liver lipids were extracted with chloroform-methanol (2:1^*v*^/_v_) according to the method described by Folch et al. [23].

### Biochemical analysis

Erythrocyte and hepatic Phospholipids was estimated using the procedure described by Stewart [24]. Triglyceride concentration was determined following the protocol described by Carr et al. [25]. Total cholesterol concentration was estimated according to the method of Allain et al. [26], and HDL-cholesterol according to Warmick et al. [27]. LDL-cholesterol was evaluated using Friedewald’s equation [28]. Aspartate aminotransferase (AST) and alanine aminotransferase (ALT) activities were determined using Randox diagnostic kits (Randox Laboratory Limited, Antrim, UK).

### Estimation of 3-hydroxy-3-methyl-glutaryl CoA (HMG-CoA) reductase activities

The ratio of the concentration of 3-hydroxymethyl-glutaryl CoA (HMG CoA) to mevalonate in the liver was used as a measure of the activity of HMG-CoA reductase [29].

#### Statistical analysis

Results are shown as mean ± SEM. Mean of the various groups were compared by using One-Way Analysis of Variance (ANOVA) followed by Turkey’s multiple comparisons. Results with a *p* < 0.05 were considered significant.

## Results

From the 2000 g powdered sample *G. lucidum* used in this study, 45.3 g GT was extracted. This translates to 2.27 g GT per 100 g *G. lucidum* powder. Body weight change was significantly higher in mice administered chloroquine alone and chloroquine in combination with 100 mg/kg Ganoderma terpenoid extract compared with infected untreated mice (Table 1). The observed difference in body weight change between mice administered chloroquine and those administered GT at 100 mg/kg was not significant. The relative liver, kidney, spleen and brain weights were not significantly different between mice administered GT (100 and 250 mg/kg) compared to chloroquine (Table 1).

**Table 1:** Body weight and relative organ weights of Plasmodium berghei-infected mice administered a combination of Ganoderma terpenoid extract and chloroquine for a period of 12 days

	CQ	GT_100_ + CQ	GT_250_ + CQ	INF-CTR
Body weight change (g)	12.3 ± 1.2^a^	10.4 ± 0.7^a,b^	8.6 ± 0.5^b^	−9.2 ± 0.2^c^
Relative organ weight				
Liver	0.08 ± 0.001^a^	0.08 ± 0.001^a^	0.08 ± 0.001^a^	0.10 ± 0.001^b^
Kidney	0.03 ± 0.001^a^	0.03 ± 0.001^a^	0.03 ± 0.001^a^	0.04 ± 0.001^a^
Spleen	0.007 ± 0.001^a^	0.007 ± 0.001^a^	0.007 ± 0.001^a^	0.013 ± 0.001^b^
Brain	0.03 ± 0.001^a^	0.03 ± 0.001^a^	0.03 ± 0.001^a^	0.03 ± 0.001^b^

Values are expressed as means ± SEM of 5 or 8 determinations. Values in the same rows carrying different superscript are statistically significant (*p* < 0.05)

**Fig. 1:**
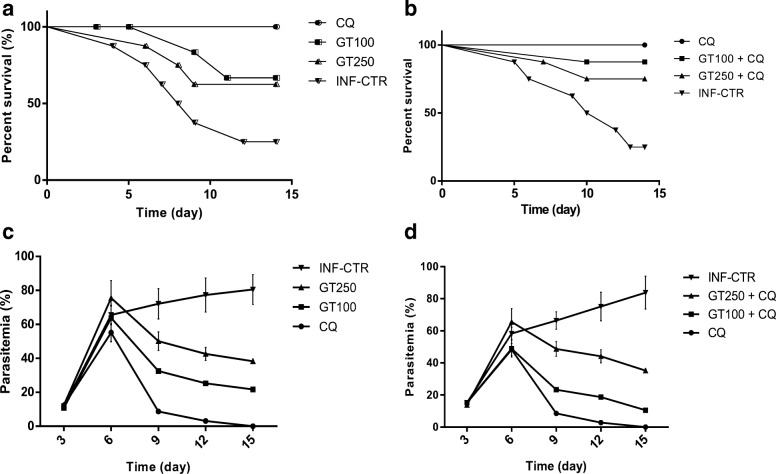
Percentage survival of *Plasmodium berghei* infected mice administered Ganoderma terpenoid extract (GT) alone (**a**) and a combination of GT plus chloroquine (**b**) with their corresponding parasitemia profile (**c**) and (**d**) respectively. Values are means ± SEM of 5 or 8 determinations

**Fig. 2:**
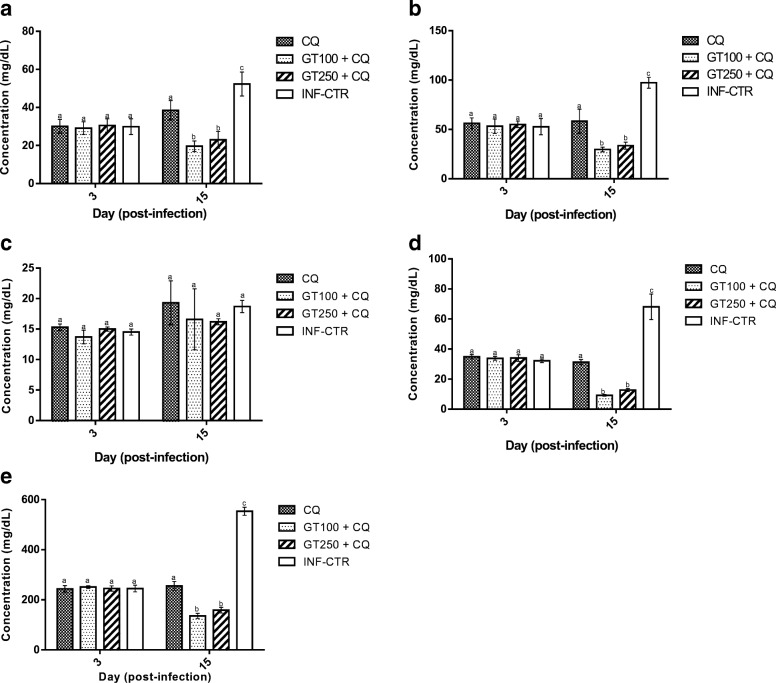
Erythrocyte triglyceride (**a**), total cholesterol (**b**), HDL-cholesterol (**c**), LDL-cholesterol (**d**) and phospholipid (**e**) of *Plasmodium berghei* infected mice administered terpenoid extract of *Ganoderma lucidum* in combination with chloroquine for a period of 12 days. Values are means ± SEM of 5 or 8 determinations. Bars carrying different alphabets are significant (*p* < 0.05)

**Fig. 3:**
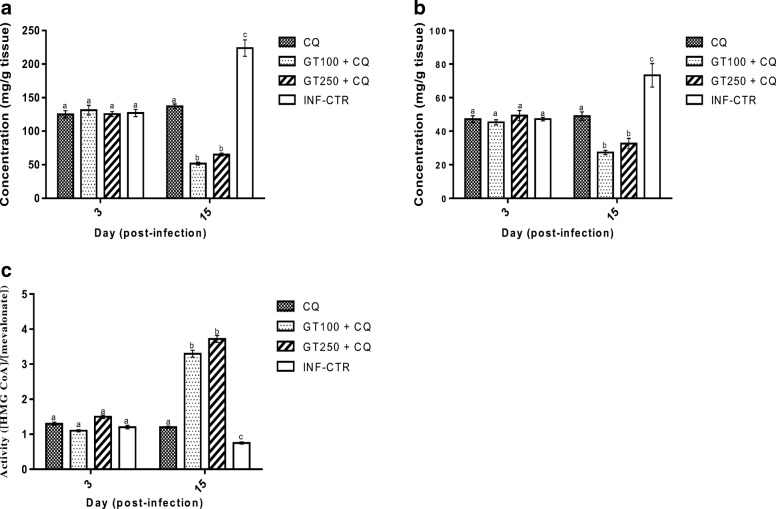
Liver total cholesterol concentration (**a**), phospholipid concentration (**b**) and HMG-CoA reductase activity (**c**) of *Plasmodium berghei* infected mice administered terpenoid extract of *Ganoderma lucidum* in combination with chloroquine for a period of 12 days. Values are means ± SEM of 5 or 8 determinations. Bars carrying different alphabets are significant (*p* < 0.05)

**Fig. 4:**
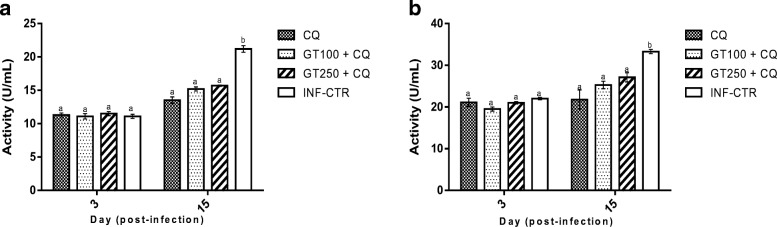
Serum aspartate aminotransferase, AST (**a**) and alanine aminotransferase, ALT (**b**) activities of *Plasmodium berghei* infected mice administered terpenoid extract of *Ganoderma lucidum* in combination with chloroquine over a period of 12 days. Values are means ± SEM of 5 or 8 determinations. Bars carrying different alphabets are significant (*p* < 0.05). Legend: Note: INF-CTR: Infected control, infected mice administered 1 mL dimethyl sulfoxide (DMSO); GT_100_: Infected mice administered Ganoderma terpenoid extract at 100 mg/kg body weight/mouse/day; GT_250_: infected mice administered Ganoderma terpenoid extract at 250 mg/kg body weight/mouse/day; CQ, Infected mice administered chloroquine at 30 mg/kg body weight/mouse/day; Note: INF-CTR: Infected control, infected mice administered 1 mL dimethyl sulfoxide (DMSO); GT_100_ + CQ: Infected mice administered a combination of Ganoderma terpenoid extract at 100 mg/kg plus 30 mg/kg chloroquine/mouse/day; GT_250_ + CQ: Infected mice administered a combination of Ganoderma terpenoid extract at 250 mg/kg plus 30 mg/kg chloroquine/mouse/day

The survival rate of the mice in response to *Plasmodium berghei* challenge was significantly increased from 66 to 87% due to combined administration of Ganoderma terpenoid extract at 100 mg/kg and CQ compared with when the same dose of the extract was administered alone and from 62 to 75% due to administration of the extract at 250 mg/kg in combination with CQ  compared with when the extract was administered alone (Fig. 1a and b, respectively). Significant reduction in percentage parasitemia from 21.7 to 10.5% due to combination of Ganoderma extract at 100 mg/kg with chloroquine (Fig. 1c and d, respectively). Erythrocyte triglyceride (Fig. 2a), total cholesterol (Fig. 2b), LDL-cholesterol (Fig. 2d) and total phospholipid (Fig. 2e) concentrations were significantly lower in GT + CQ-treated mice compared to infected but untreated mice (INF-CTR). No significant alteration was observed in erythrocyte HDL-cholesterol level between the treated mice groups and the untreated mice (Fig. 2c). Liver cholesterol (Fig. 3a) and Phospholipid (Fig. 3b) concentrations were significantly lower in mice administered GT + CQ compared to infected but untreated mice. 3-Hydroxymethylglutaryl-CoA reductase (HMG-CoA reductase) activity expressed as a ratio of [HMG-CoA]/[mevalonate] was significantly higher mice administered Ganoderma terpenoid extract (100 and 250 mg/kg) in combination with CQ compared to those administered CQ alone and the infected but untreated mice (Fig. 3c). Serum aspartate aminotransferase (AST) (Fig. 4a) and alanine aminotransferase (ALT) (Fig. 4b) activities were significantly lower in GT + CQ-treated and CQ-treated mice groups compared to infected but untreated mice.

## Discussion

*Ganoderma lucidum* has been consumed for several years in countries such as Japan, China and North Korea due to its many recognized health benefits [30]. In Nigeria and her neighboring West African states, mushroom are consumed for their nutritional benefit rather than for their medicinal values. The component parts of this mushroom including the mycelium, fruit bodies and spores have been demonstrated to exhibit different bioactive properties including antioxidant [15], antitumor [9], anti-inflammatory [10], antidiabetic [31], antimicrobial and antimalarial [12–14] activities, and they are currently being deployed in the management of diseases such as cancer hypertension), immunosuppression and hypercholesterolemia [32]. Based on the positive attributes of mushroom consumption on animal nutrition and health, increase awareness on the inclusion of mushroom in our daily meal is warranted. As such, research data demonstrating the potential attributes of these mushrooms in animal and human studies will go a long in achieving this set goal.

The 2.27% yield of GT obtained in this study is higher compared to 1.67% reported by Gao et al. [21]. The present study, demonstrated the anti-plasmodial activity of Ganoderma terpenoid extract administered either singly or in combination with chloroquine in enhancing the survival rate of mice infected with *P. berghei* in addition to drastically inhibiting the parasite growth and survival in mice. Significant improvement in body weight observed in the infected mice administered Ganoderma terpenoid extract in combination with chloroquine further attested to the positive health status of the animal. The efficacy of triterpenes extracted from different fungi as potential anti-plasmodial agents has been reported by several authors. In a study by Goulart et al. [33], terpenoid related molecules such as farnesol, nerolidol, limonene and linalool were demonstrated to inhibit the proliferation of *P. falciparum* in vitro at the intraerythrocytic stages. In a similar study, Jordão et al. [34] demonstrated that nerolidol inhibits the de novo synthesis of the isoprenic chain attached to the benzoquinone ring during the intraerythrocytic stages of *P. falciparum*. Limonene, a triterpene was also reported to inhibits protein isoprenylation in the same stages of the parasite [35]. Halogenated monoterpenes extracted from the marine red alga, *Plocamium cornutum* (Plocamiaceae) exhibited significant anti-plasmodial activity against a chloroquine-sensitive strain of *P. falciparum* [36]. Similarly, the eremophilane sesquiterpenoids, berkleasmins A and C, isolated from *Berkleasminum nigroapicale*, were reported to exhibit in vitro antiplasmodial activity with IC_50_ values of 6.0 and 5.4 μg, respectively [37]. Lanostanes isolated from the ethyl acetate extract of *Ganoderma lucidum* was reported to exhibit in vitro anti-plasmodial activity with IC_50_ values of between 6 to 20 μM [38]. In a separate study, garcihombronane D, another lanostane from *Garcinia cymosa* (Clusiaceae) showed selective activity against *P. falciparum* with IC_50_ of 7.7 μM) [39].

Results from this study showed that erythrocytes lipids including triglycerides, cholesterol, LDL-cholesterol and phospholipid were significantly lower in infected mice administered Ganoderma terpenoid extract in combination with chloroquine compared with infected but untreated mice. The observation that this trend was not repeated in mice treated with chloroquine alone seems to suggest that the this hypolipidemic action could be attributed to Ganoderma terpenoid extract. Several studies have reported the hypolipidemic activity of different extracts of Ganoderma species. Oluba et al. [12] had earlier observed a positive correlation between serum and liver lipoprotein cholesterol concentration and parasitemia level in *Plasmodium berghei* infected mice treated with aqueous extract of *Ganoderma lucidum*. The malaria parasites have a high requirement for cholesterol and phospholipids for its survival in the human host [40]. Circulating HDL-cholesterol particles and erythrocytic membrane are the potential sources of cholesterol and phospholipids for these parasites [40]. Erythrocyte phospholipids content has been demonstrated to increase 500 folds following malarial infection [41]. During the late stage of the parasite development, infected erythrocytes contain 3–5 times more phospholipids than uninfected cells [17]. Vial et al. [42] also reported that the infected erythrocytes contain phospholipid synthesizing enzymes. Thus, potent inhibitors of plasmodial phospholipid synthesis was previously characterized as potential target for anti-malarial chemotherapy due to its crucial role to the parasite survival [19].

It was also important to evaluate the total cholesterol and phospholipid contents of the liver because the exoerythrocytic stage of the malaria parasite life cycle occurs in the liver. It has been shown that therapeutic agents targeted at this stage of the parasite life cycle will go a long way in preventing clinical episodes of malaria. Results from this study showed that Ganoderma terpenoid extract does not only exert its hypolipidemic action in the erythrocytes but also in the liver. Liver total cholesterol and phospholipid contents were significantly reduced due to administration of the extract in *P. berghei* infected mice. Similar to what obtained in the erythrocyte, chloroquine administration does not affect liver phospholipid and total cholesterol concentrations. Thus, suggesting that chloroquine may be acting through other mechanisms other than reduction in hepatic lipids. In addition, hepatic HMG-CoA reductase, the rate limiting enzyme in de novo cholesterol synthesis measured as a ratio of HMG-CoA concentration to mevalonate concentration was significantly higher in the extract treated mice. This is indicative of the fact that the extract inhibits the conversion of the substrate, HMG-CoA to mevalonate by the enzyme HMG-CoA reductase. That is the extract inhibits hepatic cholesterogenesis. This observation agrees with the report of Wang et al. [43] who had earlier demonstrated that lanostane triterpenes isolated from the fruit bodies of *Ganoderma leucocontextum* from Tibet, inhibited HMG-CoA reductase activity in vitro in rat pig microsomes. In another study, Hajjaj et al. [44] reported that 26-oxygenosterols isolated from *G. lucidum* inhibited the enzyme lanosterol 14α-demethylase which catalyzes the conversion of 24,25-dihydrolanosterol to cholesterol in human hepatic cell lines.

In order to ascertain the potential toxicity of Ganoderma terpenoid extract used in this study, serum AST and ALT activities were measured. Data obtained showed that mice administered Ganoderma terpenoid extract had significantly lower AST and ALT activities compared with untreated control. The enzymes AST and ALT are membrane-bound enzymes and are routinely used as biomarkers to estimate the extent of damage to the liver. Increase levels of this enzymes in the serum or plasma is indicative of potential damage to hepatic cells thus bringing about their leakage to the plasma. Thus, high serum AST and ALT activities are recognized markers of cellular damage and functional integrity of liver cell membrane [45, 46]. In the present study, Ganoderma terpenoid extract was observed to protect the liver against *P. berghei*-induced damage. This is demonstrated in the significant reduction in serum AST and ALT activities in *P. berghei*-infected mice treated with the GT extract. In addition, the relative liver, kidney, spleen and brain weights of mice treated with the extract was not different from that obtained for chloroquine-treated mice. This observation is in agreement with the reports by several authors. Oluba et al. [12, 14] reported that crude aqueous and ethanolic extracts of *G. lucidum* showed hepatoprotection against *P. berghei*-infected mice. Similarly, Wu et al. [47] showed that Ganoderma triterpenoids demonstrated hepatoprotection against oxidative damage induced by *tert*-butyl hydroperoxide in human hepatic HepG2 cells.

## Conclusion

This study demonstrated the antiplasmodial activity of Ganoderma terpenoid extract through its reduction in parasitemia and thus improved survival rate in mice infected with *P. berghei*. It was also demonstrated that chloroquine potentiates the curative effect of Ganoderma terpenoid extract on *P. berghei* in mice. Furthermore, Ganoderma terpenoid extract exhibited significant hypolipidemic effect through reduction in infected erythrocyte lipids (triglycerides, total cholesterol, LDL-cholesterol and phospholipid) and this is suggested as a possible mechanism for its anti-plasmodial activity.

## Abbreviations

ALT: Alanine aminotransferase
AST: Aspartate aminotransferase
CQ: Chloroquine
DMSO: Dimethylsulfoxide
GT: Ganoderma terpenoid extract
HDL: High-density lipoprotein
HMG-CoA: 3-hydroxy 3-methylglutaryl-CoA
IMRAT: Institute for Advanced Medical Research and Training
INF-CTR: Infected control
LDL: Low-density lipoprotein
PBS: Phosphate buffered saline
UCH: University College Hospital
